# Potential Physiological Parameters to Indicate Inner States in Dogs: The Analysis of ECG, and Respiratory Signal During Different Sleep Phases

**DOI:** 10.3389/fnbeh.2019.00207

**Published:** 2019-09-13

**Authors:** Anna Bálint, Huba Eleőd, János Körmendi, Róbert Bódizs, Vivien Reicher, Márta Gácsi

**Affiliations:** ^1^Department of Ethology, Eötvös Loránd University, Budapest, Hungary; ^2^MTA-ELTE Comparative Ethology Research Group, Budapest, Hungary; ^3^Institute of Health Promotion and Sport Sciences, Faculty of Education and Psychology, Eötvös Loránd University, Budapest, Hungary; ^4^Institute of Behavioral Sciences, Semmelweis University, Budapest, Hungary; ^5^National Institute of Clinical Neuroscience, Juhász Pál Epilepsy Centrum, Budapest, Hungary

**Keywords:** dog, polysomnography, sleep, electroencephalography, electrocardiography

## Abstract

The sleeping activity of family dogs has been studied increasingly in the past years. Recently, a validated, non-invasive polysomnographic method has been developed for dogs, enabling the parallel recording of several neurophysiological signals on non-anesthetized family dogs, including brain activity (EEG), eye movements (EOG), cardiac (ECG), and respiratory activity (PNG). In this study, we examined the ECG (*N* = 30) and respiratory signals (*N* = 19) of dogs during a 3-h sleep period in the afternoon, under laboratory conditions. We calculated four time-domain heart rate variables [mean heart rate (HR), SDNN, RMSSD, and pNN50] from the ECG and the estimated average respiratory frequency from the respiratory signal. We analyzed how these variables are affected by the different sleep-wake phases (wakefulness, drowsiness, NREM, and REM) as well as the dogs’ sex, age and weight. We have found that the sleep-wake phase had a significant effect on all measured cardiac parameters. In the wake phase, the mean HR was higher than in all other phases, while SDNN, RMSSD, and pNN50 were lower than in all other sleep phases. In drowsiness, mean HR was higher compared to NREM and REM phases, while SDNN and RMSSD was lower compared to NREM and REM phases. In REM, SDNN, and RMSSD was higher than in NREM. However, the sleep-wake phase had no effect on the estimated average respiratory frequency of dogs. The dogs’ sex, age and weight had no effect on any of the investigated variables. This study represents a detailed analysis of the cardiac and respiratory activity of dogs during sleep. Since variations in these physiological signals reflect the dynamics of autonomic functions, a more detailed understanding of their changes may help us to gain a better understanding of the internal/emotional processes of dogs in response to different conditions of external stimuli. As such, our results are important since they are directly comparable to human findings and may also serve as a potential basis for future studies on dogs.

## Introduction

Dogs (*Canis familiaris*) have been proposed as a suitable model for studying the evolution of human cognition and social cognitive processes due to their evolutionary adaptation to the human environment and human-analog socio-cognitive skills (behavioral: Miklósi and Topál, 2013; neural mechanisms: Bunford et al., 2017). Considering our shared history dating back at least 14000 years (Savolainen et al., 2002; Miklósi, 2008), dogs represent the main subject of studies concentrating on the relationship between humans and animals (Hosey and Melfi, 2014).

The human-animal bond is characterized as a mutually beneficial and dynamic relationship between people and animals (Hosey and Melfi, 2014) and there has been a lot of studies concentrating on the effect of dogs on humans. Adopting a pet dog has been found to be associated with improved health status, lower risk of certain heart diseases and fewer visits to the doctor. Even being in the presence of a dog had short-term effects, affecting cardiovascular and psychological indicators of arousal (for a review see Friedmann, 2006). On the other hand, we still don’t have much information about the effect of humans on the physiological and neurobiological dynamics of dogs.

Kis et al. (2014) developed a non-invasive polysomnographic method that can be applied to family dogs without prior training or any special requirements. Based on this method, the neurophysiological signals of dogs during a 3-h sleep were recorded (e.g., Kis et al., 2017a, b; Bunford et al., 2018), measuring EEG, EOG, ECG, and respiratory signals. However, until now, only the EEG signals were comprehensively analyzed. Since the technique has been developed following human polysomnographic methods, it also allows for a more direct comparison of experimental results with humans.

The sleep process is characterized by the activation of a number of neural circuits, which cooperate in order to control sleep according to hormonal changes, local factors such as adenosine accumulation, circadian variations and other unknown factors (Saper et al., 2005). One of the key roles in the physiology of sleep is played by the autonomic nervous system (ANS), whose regulation modulates cardiorespiratory functions during sleep onset and the transition to different sleep stages (Tobaldini et al., 2013). The two branches of the ANS, sympathetic and parasympathetic nervous system, regulate visceral functions in order to maintain the homeostatic milieu of the body and to render the body able to react and to adapt to external and internal stressor stimuli (Montano et al., 2009; Tobaldini et al., 2013).

It is well known that during sleep, autonomic cardiac control fluctuates between sympathetic and parasympathetic predominance, mainly according to the transition to different sleep stages [wakefulness, REM (i.e., rapid eye movement) and NREM (i.e., non-rapid eye movement)]. Somers et al. (1993) showed that the cardiovascular system is strongly affected by the sleep stages in humans. For example, from N1 to N3, a gradual decrease is observed in HR, blood pressure, breathing frequency and muscle sympathetic nerve activity, with minimum values reached during N3, also called “quiet sleep.” REM sleep, however, is characterized by an opposite behavior, with a sort of “activation” of cardiovascular system to levels sometimes higher than wakefulness (Tobaldini et al., 2013), accelerated and irregular respiratory rhythm and absence of muscle tone, although occasionally limb movements and jerks occur (Dauvilliers et al., 2007).

In the earlier literature, one of the most common model animals to study the physiological changes during sleep has been the cat (e.g., see Coote, 1982). Although these investigations have greatly extended our general knowledge on the cardiorespiratory changes in mammals in different sleep phases, it is important to note that they have been conducted under very specific conditions using chronically implanted electrodes on laboratory housed animals.

Although in humans, cardiac activity is largely modulated through reflex loops and hypothalamic-brainstem centers, the “central autonomic network” appears to be responsible for rapid changes in behavior-related autonomic activity, particularly in the sensory, emotional, and cognitive dimensions. The highest levels of sensory and emotional information are integrated by autonomic cardiac activity (Appelhans and Luecken, 2006). For example, a state of physiological arousal caused by psychological stress is characterized by an increased HR, while a decrease can be seen in a stable, calm psychological state. The flexible variation of the HR or heart rate variability (HRV) is widely considered as a reliable tool to evaluate cardiovascular autonomic control.

In humans, notable relationships have been shown between autonomic functions and various diseases and mental states, including cardiac dysfunction, hypertension or psychiatric disorders. Analysis of HRV has additionally been used as an indicator of acute and chronic stress, mental challenges and emotional states. HRV may also be influenced by many physiological factors, such as gender, age, circadian rhythm, respiration or body position (Sztajzel, 2004).

In recent times, veterinary science and behavioral research has also adopted the analysis of HR and HRV, examining autonomic dynamics from various perspectives including pathological conditions, stress, behavioral dysfunction, temperament or emotional states in a number of different animal species, including pigs, cattle, horses, sheep, and goats (e.g., von Borell et al., 2007).

In dogs, HR and HRV changes have been investigated from many different perspectives as well. It has been shown that physical activity increased HR, while different body positions and walking did not have an effect on the HRV (Maros et al., 2008). In the same study, the attentive state of dogs was found to significantly increase the HRV, while causing a distinct individual HR change as well. Finally, during separation from the owner, the HR did not increase, but showed a significant increase when a stranger was petting the dog (Maros et al., 2008). Another study found similar results regarding the HR during separation from the owner, accompanied by an increase in the HRV (Gácsi et al., 2013). However, in a situation where the dogs were approached by a human in a threatening manner, the HR increased significantly, while the HRV decreased (Gácsi et al., 2013). Age has also been shown to affect HRV in dogs. Takeuchi and Harada (2002) have found that the spectral density of HRV showed age related differences, with the ratio of the low frequency and high frequency components of HRV (LF/HF ratio) showing lower amplitudes throughout the day in older dogs.

In humans, gender-specific differences in autonomic activity have also been shown to affect different HRV parameters (Agelink et al., 2001). Interestingly, in dogs, no sex differences were found in the ECG parameters (Hanton and Rabemampianina, 2006) or in the mean HR and HRV parameters (Maros et al., 2008). In the case of dogs, weight has been often cited as an important factor determining HR (e.g., Boon et al., 1983; Lombard, 1984), however, other studies have contradicted this result by demonstrating no correlation between HR and body weight (e.g., Lamb et al., 2010).

Respiration is controlled by complex neural networks in the brain. Although the most important regulatory inputs involve reflex mechanisms, respiration is also influenced by internal and external environmental changes. In humans, autonomic breathing is not only controlled by metabolic demands but also constantly responds to changes in emotions, such as sadness, happiness, anxiety, and fear. Thus, the final respiratory output involves a complex interaction between the brainstem and higher centers (Homma and Masaoka, 2008). For instance, in humans, more rapid breathing has been shown during an arousal state (Nyklíček et al., 1997; Boiten, 1998), and respiratory changes have been observed in response to natural noises, unpleasant sounds or photographs with emotional content (Boiten et al., 1994; Masaoka and Homma, 1997; Gomez and Danuser, 2004). Personality differences may also cause different respiratory patterns during mental stress and physical load (Masaoka and Homma, 1997). Respiration undergoes important modifications during sleep, becoming deeper and more regular with the synchronization of sleep (deep sleep) and shallower and more frequent during REM sleep (Lanfranchi et al., 2007).

In contrast to humans, and in contrast to HR and HRV, curiously little is known about the respiratory changes of dogs under different physiological or emotional conditions. An invasive physiological study described similar changes in dogs than in humans during sleep: in NREM, compared to the wake state, the ventilatory volume decreased, while REM sleep has been characterized by more rapid, shallow, and irregular breathing, with an increased ventilatory volume (Phillipson et al., 1976). Another, non-invasive study did not find a meaningful difference between the resting and sleeping respiratory rate of dogs (Rishniw et al., 2012), although they did not investigate separate sleep phases. Taken together, we still lack a more detailed understanding of how respiratory rate and respiratory patterns change under different conditions in dogs.

In this study, our aim is to present an analysis of the cardiorespiratory activity of dogs during different sleep-wake stages, reflecting the varying ANS activity of dogs during sleep and wake. In separate analyses, we examine the effects the dogs’ sex, age and weight on the mean HR, the three different time domain HRV parameters (SDNN, RMSSD, pNN50: see section “Materials and Methods”), and the estimated average respiratory frequency of dogs in each sleep-wake phase.

## Materials and Methods

### Ethics Statement

Owners were recruited from the Family Dog Project (Eötvös Loránd University, Department of Ethology) database, they participated in the study without monetary compensation and provided their informed consent before the onset of the experiment.

The research was carried out in accordance with the Hungarian regulations on animal experimentation and the Guidelines for the Use of Animals in Research described by the Association for the Study of Animal Behavior (ASAB). All experimental protocols were approved by the Scientific Ethics Committee for Animal Experimentation of Budapest, Hungary (No. of approval: PE/EA/853-2/2016).

### Subjects

Subjects were *N* = 30 family dogs (16 male, 14 female) with the average age of 7.1 years (minimum = 1.3, maximum = 15.7 years), from different breeds (see Table 1). Participating in the sleep study did not require prior training, the only criteria was to surpass the age of 1 year. The average weight was 21.3 kg (minimum = 3.5, maximum = 42 kg).

**TABLE 1:** Information on the subject dogs.

**ID**	**Breed**	**Sex**	**Age**	**Weight (kg)**	**Sleep efficiency (ratio of wake compared to all other sleep phases; %)**	**Ratio of each phase during sleep measurement: drows/NREM/REM (%)**	**Starting time of 3-hour long sleep measurement**
Dog 1	border collie	male	1.3	17	45.41	62.28/17.10/20.62	14:30
Dog 2	Australian shepherd	male	2.2	24.5	41.72	60.63/32.57/6.8	15:24
Dog 3	golden retriever	female	2.5	25	89.21	9.38/56.68/33.94	14:00
Dog 4	golden retriever	female	2.5	28	79.51	38.20/22.87/38.93	10:30
Dog 5	golden retriever	male	2.9	28	62.22	48.56/45.68/5.76	15:02
Dog 6	golden retriever	male	2.9	34	86.1	30.80/26.24/42.96	17:30
Dog 7	bichon Havanese	male	3.2	3.5	91.98	6.36/65.40/28.24	14:47
Dog 8	mix	female	3.8	23.3	35.3	87.91/9.34/2.75	17:07
Dog 9	mix	female	4.5	22	69.88	32.73/50.25/17.02	17:30
Dog 10	white Swiss shepherd	female	4.5	25	47.32	51.56/46.48/2.81	19:17
Dog 11	English cocker spaniel	male	4.6	14.7	18.37	51.45/45.63/2.92	19:12
Dog 12	border collie	female	5.1	16	93.74	27.30/42.23/30.47	19:06
Dog 13	mix	female	5.3	30	74.34	56.43/28.71/14.86	15:42
Dog 14	mix	female	5.7	10	64.4	22.94/65.88/11.18	19:20
Dog 15	boxer	male	6	32	66.14	67.49/11.01/21.5	10:29
Dog 16	mix	male	7.3	13.5	86.1	22.97/51.48/25.55	19:08
Dog 17	mix	male	8.7	42	57.22	78.16/16.45/5.39	15:49
Dog 18	mix	female	8.9	10.9	90.99	20.48/38.33/41.19	14:33
Dog 19	border collie	female	8.9	21	78.26	33.09/36.61/30.3	13:49
Dog 20	German shepherd	male	9.2	32.8	91.67	23.14/47.72/29.14	15:55
Dog 21	mix	female	9.7	18.5	79.86	30.52/57.85/11.63	15:45
Dog 22	schipperke	male	10.2	5	55.98	65.21/21.07/13.72	18:40
Dog 23	puli	male	10.7	12.8	76.71	18.06/40.71/41.23	17:21
Dog 24	mix	female	10.8	27	83.72	26.69/54.97/18.34	14:49
Dog 25	whippet	male	10.9	13	76.43	81.68/4.57/13.75	13:32
Dog 26	mix	female	11	14	34.32	26.26/54.54/19.2	14:32
Dog 27	Tibetan terrier	male	11.4	12.2	36.37	23.95/61.45/14.6	15:18
Dog 28	Magyar vizsla	female	13.5	21.2	81.47	30.11/56.09/13.8	19:39
Dog 29	mix	male	15.7	27	82.69	20.71/43.87/35.42	17:42
Dog 30	golden retriever	male	8.6	35	82.58	14.67/38.07/47.26	16:40

### General Procedure

**FIGURE 1:**
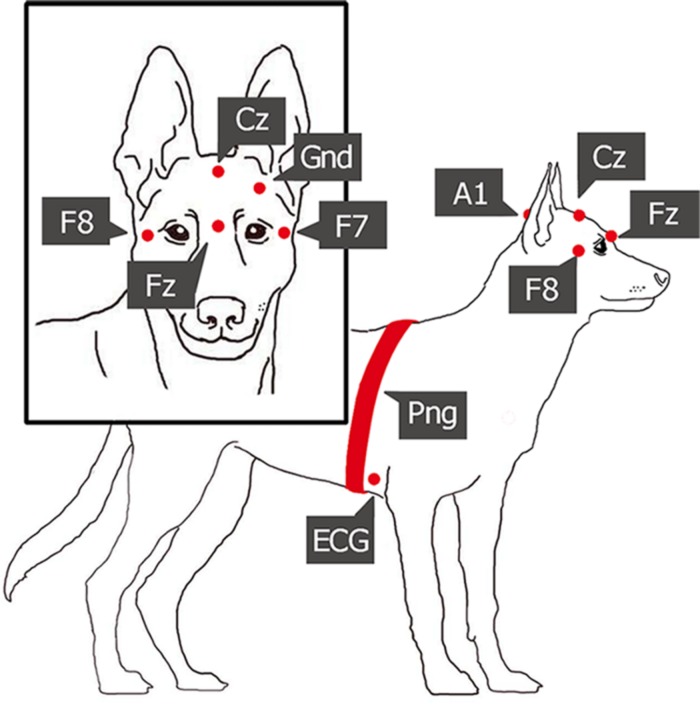
Placement of the electrodes and the respiratory band (Fz-Cz, frontal and central midline; F7-F8, right and left electrodes placed on the zygomatic arch, next to the eyes; A1, reference electrode; Gnd, ground electrode; ECG, electrocardiographic electrode; Png, respiratory band).

The measurements were performed at the Department of Ethology, University of ELTE, Budapest and the Research Center for Natural Sciences, Institute of Cognitive Neuroscience and Psychology, Budapest, in fully equipped EEG and polysomnography measurement laboratories. All the recordings were conducted during the afternoon, starting between 12 and 8 pm, except for two measurements that were recorded between 10.30 and 13.30. Before the sleep experiment started, the research staff explained the process of the measurement while the dog explored the room (5–10 min). After that, the owners settled on a mattress (provided for the sleep experiment) and held the dog’s head gently while the research staff placed the surface electrodes and the respiratory band on the dogs’ head and body (see Figure 1). During electrode placement, the dogs were positively reinforced by social (e.g., petting, praise) and/or food rewards. After checking the recorded signals, owners were asked to turn off their cell phones and engage in a quiet activity during the measurement (e.g., reading, watching a movie with earphones, sleeping). The research staff left the room and monitored the measurement on a laptop in an adjacent room. In case of electrode-malfunctioning, the experimenter entered the sleep laboratory and replaced or changed the electrode. The experimenter prepared notes about all measurements, noting all incidences (e.g., electrode-malfunctions, external disturbing factors such as noise from the hallway, occasions and causes of re-entrances) that occurred during the 3-h recordings.

### Polysomnography

**FIGURE 2:**
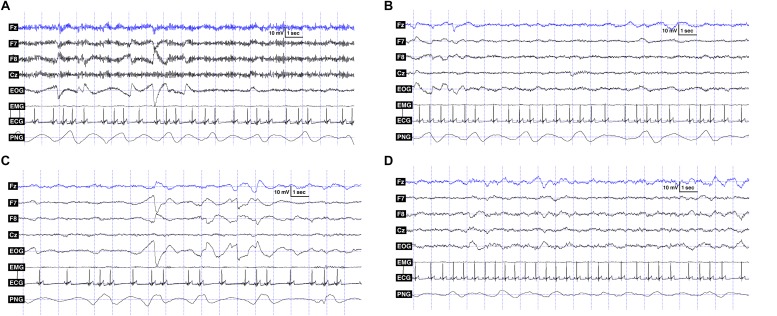
**(A)** Polysomnography data showing the recorded signals: EEG channels (Fz, F7, F8, Cz), EOG: F7 referred to F8, ECG and PNG (respiratory signal) in the wake phase. **(B)** Polysomnography data showing the recorded signals: EEG channels (Fz, F7, F8, Cz), EOG: F7 referred to F8, ECG and PNG (respiratory signal) in the drowsiness phase. **(C)** Polysomnography data showing the recorded signals: EEG channels (Fz, F7, F8, Cz), EOG: F7 referred to F8, ECG and PNG (respiratory signal) in REM phase. **(D)** Polysomnography data showing the recorded signals: EEG channels (Fz, F7, F8, Cz), EOG: F7 referred to F8, ECG and PNG (respiratory signal) in NREM phase.

Polysomnography (PSG) is a type of sleep study that allows the parallel recording of many different physiological signals from brain activity (EEG) through eye movements (EOG), to electrocardiogram (ECG), and respiration (PNG) (Ibáñez et al., 2018). In this study, we followed the previously validated PSG method on dogs (Kis et al., 2014). The only innovation we added is another electrode on the right zygomatic arch, so with this new setup, four EEG channels and two eye movement channels could be recorded instead of one. The two electrodes placed on the right and left zygomatic arch next to the eyes (F8, F7) and the scalp electrodes over the anteroposterior midline of the skull (Fz, Cz) were referred to the A1, a reference electrode which was in the posterior midline of the skull (occiput; external occipital protuberance). The ground electrode (Gnd) was attached to the left musculus temporalis. ECG electrodes were placed bilaterally over the second rib. See Figure 1 for detailed drawing of a dog with the name and exact placement of the electrodes and Figure 2 for examples of polysomnography data from the four different sleep stages.

For the recordings, gold-coated Ag/AgCl electrodes were used, secured by EC2 Grass Electrode Cream (Grass Technologies, United States). The impedance values of the EEG electrodes were kept under 20 kΩ during the recordings. The signals were collected, pre-filtered, amplified and digitized either at a sampling rate of 1024 Hz/channel, using the 25 channel SAM 25R EEG System (Micromed, Mogliano Veneto, Italy), and the System Plus Evolution software with second-order filters at 0.016 Hz (high pass) and 70 Hz (low pass), or at a sampling rate of 249 Hz/channel, using a 30-channel Flat Style SLEEP La Mont Headbox with implemented second order filters at 0.5 Hz (high pass) and 70 Hz (lowpass) as well as the HBX32-SLP 32 channel preamplifier (LaMontMedical Inc., United States).

### Data Analysis

Sleep-wake phases in the sleep recordings were visually scored in accordance with standard criteria (Rechtschaffen and Kales, 1968), adapted for dogs (Kis et al., 2014), using a program developed by Ferenc Gombos (Fercio’s EEG Plus, 2009–2018) to analyze and export data. The recordings were scored by the step-by-step analysis of 20 s epochs. Following the established conventions in Kis et al. (2014), the following sleep-wake phases were recognized, based on the given criteria: (1) wakefulness stage: fast activity in the EEG (Fz–Cz derivation), high amplitude and frequency eye movements in the EOG (LOC–Cz derivation); (2) drowsiness stage: fast EEG activity in the EEG channel (Fz–Cz derivation), accompanied by decreased amplitude and frequency eye movements in the EOG (LOC–Fz derivation), fairly regular respiration (Rsp channel); (3) Slow wave sleep (NREM): the occurrence of ≥15 μV delta (1–4 Hz) activity and/or sleep spindles (waves with 12–16 Hz frequency and ≥0.5 s duration) in the EEG (Fz–Cz derivation), no or low amplitude eye movements in the EOG (LOC–Fz derivation), relatively regular respiration (Rsp channel); and (4) REM sleep: the occurrence of rapid eye movements in the EOG (LOC–Fz derivation)—also seen as artifacts in the EEG (Fz–Cz derivation)—fast EEG activity (Fz–Cz derivation), irregular respiration (Rsp channel) and heart beats (ECG).

From our database we have selected 30 recordings of good quality, i.e.: (1) no disturbance occurred during the 3-h-long sleep (electrode malfunctioning or external noise); (2) dogs went through the whole sleep cycle, entering drowsiness, NREM and REM. In 11 recordings, the respiratory signals contained excessive amounts of noise, probably due to the progressive loosening or movement of the respiratory band. Thus, in sum, we had 30 ECG and 19 respiratory recordings.

By inspecting the durations of the alternating sleep-wake phases in the measurements, we found that the maximum length of data segments we could use in our calculations is 180 s. Thus, we segmented both the heart and respiratory data into 180 s long periods, separately in each sleep-wake phase and performed all calculations on these 180 s long segments. Then, we averaged the results of the data segments for each sleep-wake phase, for each dog, and we used these in the statistical analyses.

Electrocardiogram signal analysis: From continuous ECG measurements, we first detected the successive R-peaks using a semi-automated method. A custom-built software (JEDF, developed by JK) detected the R-peaks automatically, which were inspected visually and corrected manually. Intervals with artifacts were also indicated and removed from the dataset. From the detected R-peaks, the RR-intervals were calculated automatically, resulting in an RR-tachogram. We then segmented the RR-tachogram into 180 s long segments for each sleep-wake phase. These segments were used to compute time-domain HRV parameters using Kubios HRV Standard software^1^. The results were then averaged for each sleep-wake phase, for each dog.

Heart rate variability may be evaluated by a number of methods. The possibly most straightforward methods are to compute the so-called time domain measures. With these methods either the HR at any point in time or the intervals between successive normal complexes are determined. The most commonly examined time–domain variables include for instance the mean HR, SDNN (standard deviation of the RR intervals), SDANN (standard deviation of the average NN interval calculated over short periods), SDNN index (in humans, the mean of the 5-min standard deviation of the NN interval calculated over 24 h), RMSSD (root mean square of the differences of successive RR intervals) or pNN50 (the proportion of pairs of successive RR intervals that differ by more than 50 ms divided by the total number of RR intervals) (Malik et al., 1996).

In this study, we investigated three different HRV measures: SDNN, RMSSD, pNN50. It is important to note that these time-domain parameters are strongly correlated with certain frequency-domain parameters. SDNN is correlated with the total spectral power of the measured time period, while RMSSD and pNN50 are correlated with the high-frequency (HF) component of the HRV, which primarily reflects parasympathetic influence on the heart due to respiratory sinus arrhythmia (Malik et al., 1996; Appelhans and Luecken, 2006). As such, SDNN expresses the overall variability, while RMSSD and pNN50 represent the short-term variations (HF component of HRV) in the RR time series.

Respiratory signal analysis: In the case of the respiratory signal, we do not yet have a software-based method that could reliably detect the number of breaths. In contrast to humans, the respiratory signals of dogs show high variability (see Figure 2), causing significant difficulties in determining the exact breathing patterns. Therefore, we performed frequency analyses on the 180 s long data segments of each sleep-wake phase of each dog, according to the following steps. We first performed zero-phase digital filtering (4 Hz, 6th order, lowpass, Butterworth) on the whole signal and then segmented the data of each sleep-wake phase into 180 s long periods. We then down-sampled recordings with 1024 Hz sampling frequency to 4 Hz and interpolated (spline interpolation) those with 249 Hz sampling frequency to 4 Hz. The 180 s long data segments for each sleep-wake phase of each dog were then demeaned and detrended and a power spectrum analysis using fft (using Hanning window function on the data) was performed on them. The results of the spectrum analysis of the 180 s long data segments were then averaged, smoothed, and the maximum of the spectrogram was located in order to estimate the average respiratory frequency for that sleep-wake phase. All computations on the respiratory signal were done using the Matlab R2014b software^2^.

To statistically test our results, we used generalized linear mixed models to test the effects of our explanatory variables on five different response variables: mean HR, SDNN, RMSSD, pNN50 and the estimated average respiratory frequency. An important property of mixed effects models is that they can handle imbalances between the data of different subjects (Lindstrom and Bates, 1990). Since dogs showed high individual variability in their sleep macrostructure (i.e., differences between their sleep efficiency and the percent of time they spent in the different sleep phases; see Table 1), this affected the amount of data we could extract from the different sleep-wake phases of our subjects. The models included the sleep-wake phase (wake, drowsiness, NREM, and REM), the dogs’ sex, age, and weight as fixed effects and the dogs’ identity as a random effect. The final model was selected using stepwise backward elimination. All statistical tests were done using IBM’s SPSS 25.0 software^3^.

## Results

**FIGURE 3:**
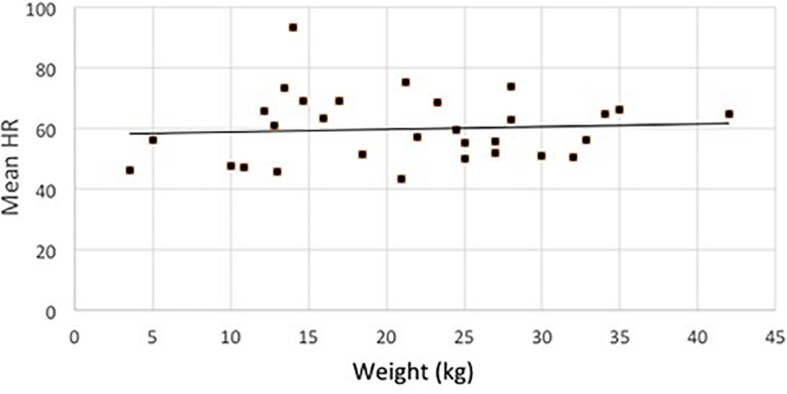
Distribution of the average HR (in all sleep-stages) of dogs in terms of their weight.

Dogs’ sex and age had no effect on any of the response variables. Interestingly, the weight of the dogs had also no effect on any of the investigated variables (see Figure 3 for HR by weight).

**FIGURE 4:**
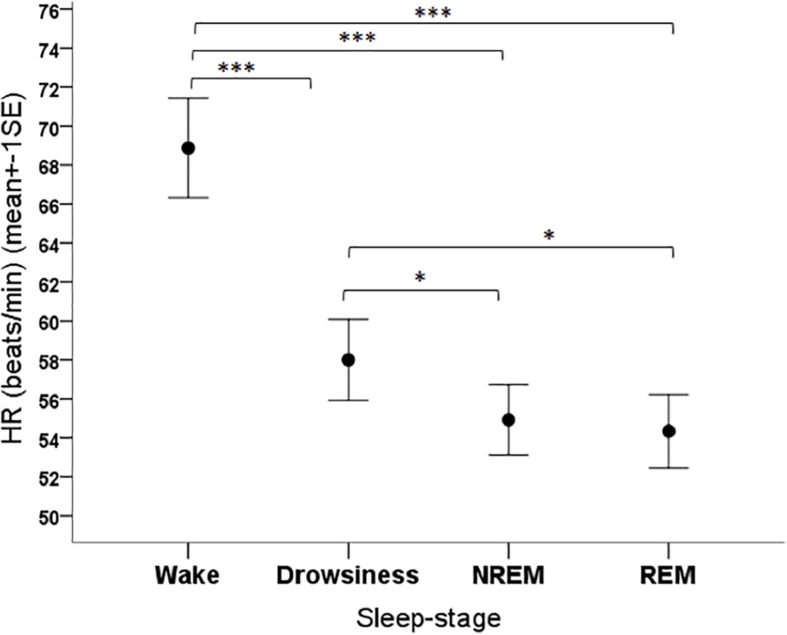
Mean heart rate of family dogs in the four sleep-stages. ^∗^*p* < 0.05, ^∗∗∗^*p* < 0.001.

The sleep-wake phase had a significant effect on all cardiac parameters. The mean HR of dogs was different depending on the phase (*F*_3__,__108_ = 22.36, *p* < 0.001), being higher in the wake phase than in all sleep phases (*p* < 0.001 for all pairwise contrasts), and it was also higher in drowsiness compared to the NREM (*p* = 0.001) and REM (*p* = 0.004) (Figure 4).

**FIGURE 5:**
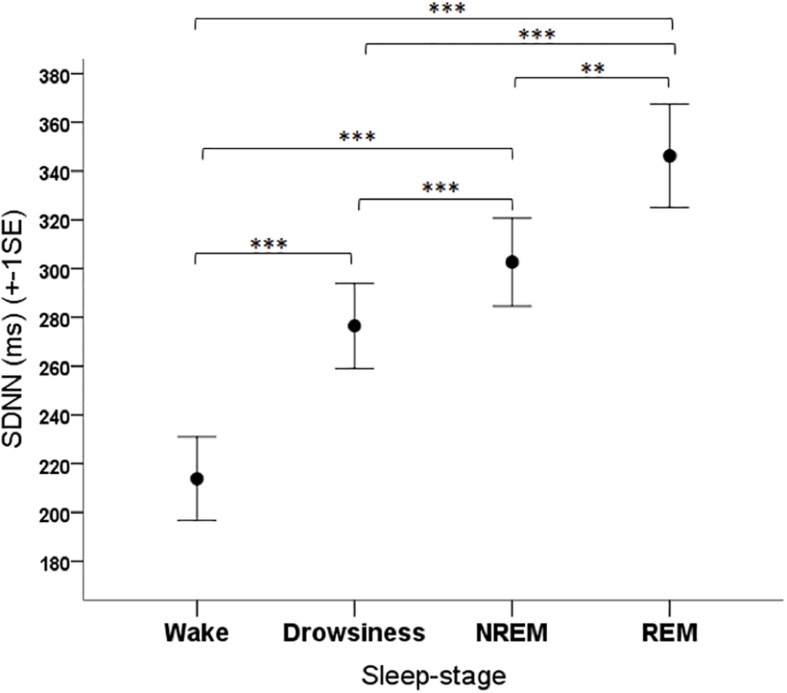
Mean SDNN parameter of family dogs in the four sleep-stages. ^∗∗^*p* < 0.01, ^∗∗∗^*p* < 0.001.

The sleep-wake phase had a significant effect on the SDNN parameter of dogs (*F*_3__,__108_ = 24.341, *p* < 0.001). Based on the *post hoc* tests, the SDNN in the wake phase was lower than in all other phases (*p* < 0.001 for all pairwise contrasts), it was higher in the REM phase compared to the NREM phase (*p* = 0.003) and to the drowsiness phase (*p* < 0.001), and it was higher in the NREM phase than in the drowsiness phase (*p* < 0.001) (Figure 5).

**FIGURE 6:**
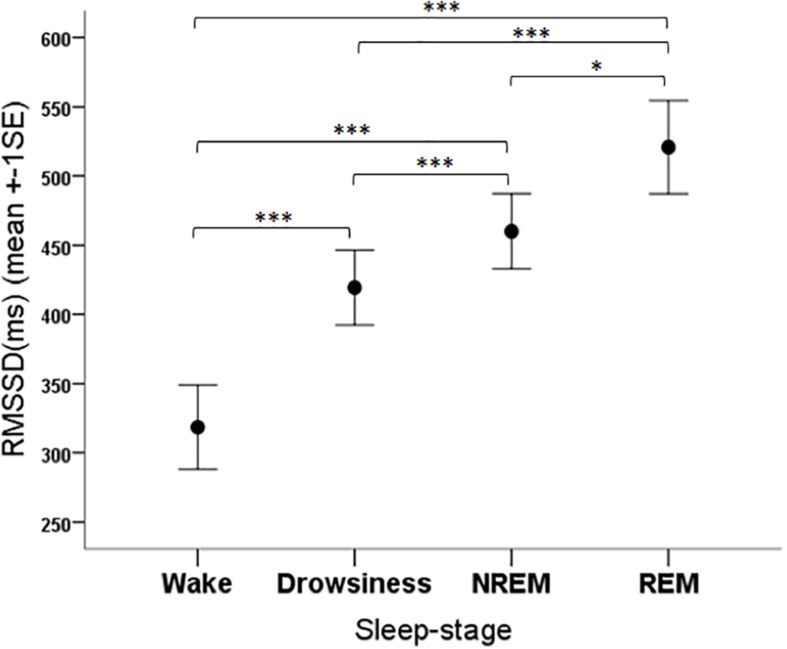
Mean RMSSD parameter of family dogs in the four sleep-stages. ^∗^*p* < 0.05, ^∗∗∗^*p* < 0.01.

The RMSSD parameter was significantly affected by the sleep-wake phase (*F*_3__,__108_ = 17.47, *p* < 0.001). The *post hoc* tests showed that the RMSSD was lower in the wake phase than in all other phases (*p* < 0.001 for all pairwise contrasts) and it was also lower in the drowsiness phase than in the NREM (*p* = 0.001) and REM (*p* < 0.001) phases, and higher in REM compared to NREM (*p* = 0.028) (Figure 6).

**FIGURE 7:**
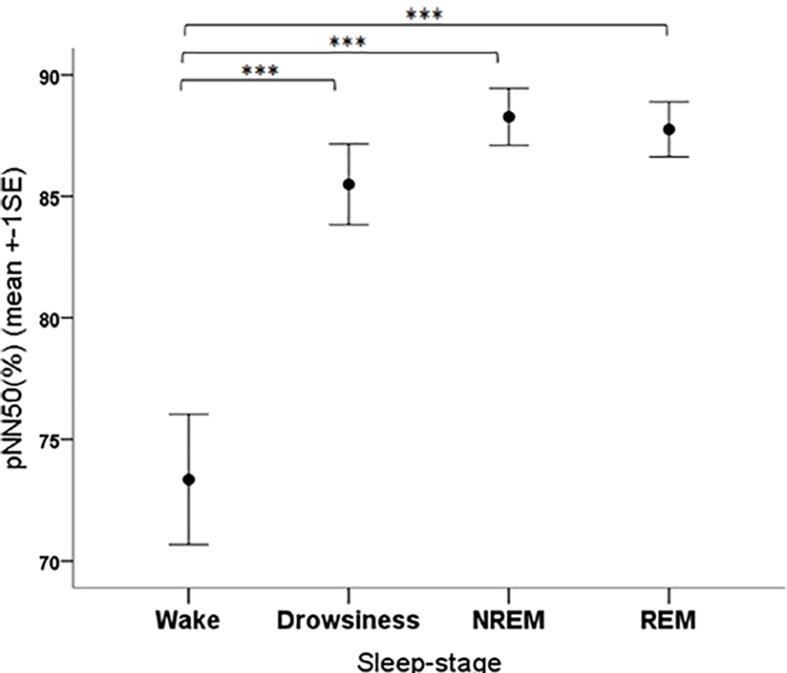
Mean pNN50 parameter of family dogs in the four sleep-stages. ^∗∗∗^*p* < 0.01.

The pNN50 parameter was significantly affected by the sleep-wake phase as well (*F*_3__,__108_ = 13.34, *p* < 0.001). The *post hoc* tests showed that the pNN50 values were significantly lower in the wake phase than in all other sleep-wake phases (all contrasts: *p* < 0.001) (Figure 7).

Neither the independent variables (sex, age, and weight) nor the sleep-wake phase had significant effects on the estimated average respiratory frequency.

## Discussion

Using a non-invasive polysomnographic method adapted for dogs, we have successfully complemented the limited knowledge we have had on the cardiorespiratory changes of dogs during sleep. Our results can be compared with other studies on dogs and two other extensively studied mammalian species, humans and cats.

Our results supported the findings of an earlier non-invasive study that investigated the cardiac changes in dogs during sleep. They measured higher HR and lower HRV (i.e., SDNN) in waking than in all other sleep stages (Varga et al., 2018). They also found that when examining the first occurrence of a sleep stage (drowsiness), it is also characterized by higher HR and lower HRV. Contrary to our results, they did not reveal differences between the REM and NREM phases, in terms of neither HR nor HRV. After social interactions, they measured differences in the dogs’ HR between the positive and negative conditions, but only in wakefulness (Varga et al., 2018).

Similarly to our findings, in both humans and cats HR has been found to decrease on entry to sleep (Coote, 1982; Baharav et al., 1995). More specifically, in humans, a number of studies have found that the HR is lower in NREM compared to the wake state (e.g., Snyder et al., 1964; Trinder et al., 2001; Coccagna and Scaglione, 2003). In REM sleep, HR becomes very variable both in humans and cats (Coote, 1982; Dickerson et al., 1993), with humans showing marked swings resulting in the lowest REM sleep levels and the highest overall sleep levels (Coote, 1982). These swings can also be seen in the cat, with a more pronounced decrease in HR during the tonic phase of REM sleep (Coote, 1982; Verrier et al., 1998) and HR surges raising the rate by as much as 26.4% (Rowe et al., 1999). Our findings in dogs are similar to the above, with the lowest average HR and the highest overall HR variability (SDNN) seen in REM sleep.

Regarding different HRV parameters, in humans, Trinder et al. (2001) have described an increase in the HF component (HF: a parameter highly correlated with RMSSD and pNN50: Malik et al., 1996; Appelhans and Luecken, 2006) during NREM sleep, while Baharav et al. (1995) found that the HF component of HRV had increased values during the whole sleep period, which is similar to our findings. The total spectrum power (a parameter strongly correlated with SDNN: Malik et al., 1996; Appelhans and Luecken, 2006) has been found to be significantly higher in REM sleep than in NREM sleep (Togo and Yamamoto, 2001), and to culminate in REM sleep during the whole sleep period (Busek et al., 2005) in humans, again in line with our findings in dogs. However, contrary to our results, human studies have found that the HF component showed a decrease in REM, while an increase in NREM sleep (Baharav et al., 1995; Vaughn et al., 1995).

In cats, similarly to our findings, the short-term variability of HR (highly correlated with RMSSD and pNN50: Appelhans and Luecken, 2006) was low in the waking state, higher in NREM than in waking, and the overall variability (highly correlated with SDNN: Appelhans and Luecken, 2006) was increased in REM compared to NREM sleep (Raetz et al., 1991). Our results, however, contradict some findings of the same study, as they found that the short-term variability appeared to be lower in REM than in NREM sleep, while the overall variability of HR was higher in waking than in NREM (Raetz et al., 1991).

While further detailed investigations are required for the explanation of such differences between species, it should be noted that the above mentioned results in cats are derived from invasive measurements on laboratory animals, using chronically implanted electrodes, whereas our measurements were conducted on family dogs, using non-invasive methodology (similarly to that of humans). Although invasive measurements undoubtedly deliver credible and solid data, the life-history and environmental/social experiences of animals participating in invasive and non-invasive measurements are markedly different, possibly even affecting their autonomic functions. Therefore, it would be important to compare the results of invasive and non-invasive dog studies and, if possible, cat studies as well, to be able to assess species differences more precisely.

Studies on humans have arrived at different conclusions regarding the relationship between gender, age, body mass index and HR/HRV parameters. For instance, Silvetti et al. (2001) have found that the SDNN values were significantly higher in males than in females, the HR decreased significantly with age, SDNN, RMSSD and pNN50 were increased in adolescents compared to children and RMSSD and pNN50 were even related to the body mass index of subjects. Antelmi et al. (2004) have found that HRV parameters decreased with age, showed gender-related differences but had no correlation with the body mass index. In dogs, studies have usually showed no differences in the ECG or HRV parameters of the two sexes (e.g., Hanton and Rabemampianina, 2006; Maros et al., 2008; Ferasin et al., 2010), while age-related changes were found in the sleep-wake rhythm (Takeuchi and Harada, 2002) and the HR of dogs (Bodey and Michell, 1996; Hezzell et al., 2013). The effect of body weight on cardiac activity of dogs is a more controversial topic, with some studies describing weight as having an inverse relationship with HR (e.g., Tilley, 1992; Kittleson and Kienle, 1998), and others a direct (e.g., Hamlin et al., 1967).

Regarding the relationship between sex and the investigated cardiac parameters, our results are in agreement with the majority of the literature, further strengthening the notion that sex hormonal differences do not seem to play a role in the variation of HR and HRV parameters in dogs. The lack of age-, and weight-related effects found in this study are, however, in contradiction with the results of some other studies on dogs (e.g., Bodey and Michell, 1996; Hezzell et al., 2013). Importantly, however, Hezzell et al. (2013) only found a positive correlation between the age and HR of dogs in a diseased population, referred to the hospital for various reasons. They could not show the same correlation in apparently healthy dogs. While the same authors have found a negative correlation (Hezzell et al., 2013), other studies (e.g., Ferasin et al., 2010; Lamb et al., 2010) have found no correlation between the HR and body weight of dogs. It is widely accepted, that HR is affected by a number of different parameters including temperament, exercise regime, diet, breed or body condition score and some authors have suggested (e.g., Lamb et al., 2010) that the lack of association between weight and HR may be due to the fact that these factors and their complex interactions outweigh the effect of weight. Accordingly, Hezzell et al. (2013) also note that, based on their analyses, it seemed that their statistical model lacked important determinants of HR. Following this logic, since the measurements in Hezzell et al. (2013)’s paper have been conducted during veterinary visits, it may reflect the fact that smaller breeds are more afraid in these vulnerable situations than larger ones. The importance of the context of HR measurements is also illustrated by the fact that while the grand average of HR under veterinary office conditions (Hezzell et al., 2013) and under laboratory conditions (being exposed to different interventions) (Behar et al., 2018) was around 124 beats/min, in two other studies, where dogs participated in a behavioral experiment with minimal physical activity and stress, the grand average of sitting/lying dogs’ HR was only around 80 beats/min (Gácsi et al., 2013) or 90–95 beats/min (Maros et al., 2008). In the present study, where dogs were resting with their owners (even less physical activity), the grand average HR was only around 69 beats/min (see Figure 5).

The lack of any findings regarding the estimated average respiratory frequency may be surprising. We cannot exclude that our estimation of the average frequency was not perfectly precise due to artifacts (e.g., originating from the ECG or sudden movements), or that the high individual variability (see Figures 2A–D) and the relatively small sample size (*N* = 19) made it more difficult to demonstrate any differences. Although some studies have found respiratory differences according to different sleep phases in dogs (Phillipson et al., 1976), humans (Hobson et al., 1965; Coote, 1982) and cats (Orem, 1980; Coote, 1982), our results are in line with some other findings in the literature. Rishniw et al. (2012) investigated the resting and sleeping respiratory rate of dogs. They found that age and body weight had no effect on the sleeping respiratory rate of dogs. Although they have not examined different sleep-stages separately, they found that the resting respiratory rate was not markedly higher than the sleeping respiratory rate, which conditions are comparable to our wake vs. sleep phases.

Heart rate variability is widely accepted to be a reliable measure of the autonomic functioning of the individual (Appelhans and Luecken, 2006; Quintana et al., 2012). The proper functioning of the ANS has a major role in emotional regulation, which is essential in the adaptation to the physical and social environment (Balzarotti et al., 2017). In humans, it facilitates decisions in challenging situations and social interactions (Appelhans and Luecken, 2006). Studies have reported that higher HRV is associated with emotional well-being, lower anxiety and better regulated emotional responding in humans (Mather and Thayer, 2018). In contrast, reduced values of HRV were found to be associated with a number of psychiatric illnesses (e.g., depression, anxiety), poor social dynamics and emotion recognition (Quintana et al., 2012). Other studies have suggested a relationship between HRV and adaptive coping strategies in humans (Appelhans and Luecken, 2006).

Heart rate variability parameters and different internal and emotional states have been shown to be associated in non-human animals as well. In calves, SDNN and RMSSD significantly decreased with increasing levels of stress load (von Borell et al., 2007). In horses, studies demonstrated a relationship between behavioral reactivity and HRV. When being handled or upon introduction of a novel object, mean HR increased while SDNN and RMSSD decreased (von Borell et al., 2007).

In dogs, increased HR has been found to be associated with emotional arousal (Zupan et al., 2016) and positive stimulation (Travain et al., 2016). Katayama et al. (2016) have found that different HRV parameters change in response to emotionally negative and positive stimuli. While SDNN decreased in positive situations, only RMSSD was lower in negative situations. The effect of emotional and behavioral regulation on HRV has also been investigated in dogs by Craig et al. (2017). They have found that dogs with bite histories had significantly lower HRV values and the owner reported aggression negatively correlated with HRV.

A study investigating the effects of human-dog interactions on the behavior and cardiac activity of dogs has found that dogs showed more appeasement gestures when the HR was increased and the RMSSD was decreased, compared to baseline values (Kuhne et al., 2014a). In a related study, the authors have found that petting and holding the dog around the head was associated with an increased SDNN. Again, HR values positively correlated with appeasement gestures. Behavioral strategies such as freezing and withdrawal showed a negative correlation with RMSSD and RMSSD/SDNN ratio (Kuhne et al., 2014b).

The diversity of results demonstrating the complex relationship between emotional states and HRV parameters clearly shows the significance of HRV in the assessment of different internal states. In this study, by analyzing the basic features of two physiological signals during a 3-h sleep period of dogs, one of our aims was to establish a groundwork for prospective studies investigating the physiological correlates of different emotional stimuli affecting dogs. For instance, in parallel studies, we aim to investigate the effects of emotionally positive vs. neutral auditory stimuli, both in awake and sleeping dogs. While it is intuitive to assume that the different emotional loadings evoke different physiological responses in dogs, by measuring physiological parameters in response to these stimuli, we can obtain a more clear-cut picture of the underlying processes. In another study, we intend to investigate the effects of two different training methods on the sleep of dogs. Again, the physiological parameters measured during their sleep will give us a more definite and coherent picture about the emotional processing of the different training techniques.

## Data Availability

The raw data supporting the conclusions of this manuscript will be made available by the authors, without undue reservation, to any qualified researcher.

## Ethics Statement

The animal study was reviewed and approved by the Scientific Ethics Committee for Animal Experimentation of Budapest, Hungary (No. of approval: PE/EA/853-2/2016). Written informed consent was obtained from the owners for the participation of their animals in this study.

## Author Contributions

AB: data processing and analysis, writing-original draft, and editing. HE: data processing, writing-editing. JK: development of applied software, data processing, supervision, and of data processing and analysis. RB: conceptualization and writing-editing. VR: data collection and scoring, and writing-editing. MG: original research idea, conceptualization, data analysis, and writing-editing.

## Conflict of Interest Statement

The authors declare that the research was conducted in the absence of any commercial or financial relationships that could be construed as a potential conflict of interest.

## Footnotes

1 https://www.kubios.com/

2 https://www.mathworks.com/

3 https://www.ibm.com/analytics/spss-statistics-software

## References

[B1] Agelink M. W. Malessa R. Baumann B. Majewski T. Akila F. Zeit T. (2001). Standardized tests of heart rate variability: normal ranges obtained from 309 healthy humans, and effects of age, gender, and heart rate. *Clin. Auton. Res.* 11 99–108. 10.1007/bf02322053 11570610

[B2] Antelmi I. De Paula R. S. Shinzato A. R. Peres C. A. Mansur A. J. Grupi C. J. (2004). Influence of age, gender, body mass index, and functional capacity on heart rate variability in a cohort of subjects without heart disease. *Am. J. Cardiol.* 93 381–385. 10.1016/j.amjcard.2003.09.065 14759400

[B3] Appelhans B. M. Luecken L. J. (2006). Heart rate variability as an index of regulated emotional responding. *Rev. Gen. Psychol.* 10 229–240. 10.1037/1089-2680.10.3.229

[B4] Baharav A. Kotagal S. Gibbons V. Rubin B. K. Pratt G. Karin J. (1995). Fluctuations in autonomic nervous activity during sleep displayed by power spectrum analysis of heart rate variability. *Neurology* 45 1183–1187. 10.1212/wnl.45.6.1183 7783886

[B5] Balzarotti S. Biassoni F. Colombo B. Ciceri M. R. (2017). Cardiac vagal control as a marker of emotion regulation in healthy adults: a review. *Biol. Psychol.* 130 54–66. 10.1016/j.biopsycho.2017.10.008 29079304

[B6] Behar J. A. Rosenberg A. A. Weiser-Bitoun I. Shemla O. Alexandrovich A. Konyukhov E. (2018). PhysioZoo: a novel open access platform for heart rate variability analysis of mammalian electrocardiographic data. *Front. Physiol.* 9:1390. 10.3389/fphys.2018.01390 30337883 PMC6180147

[B7] Bodey A. R. Michell A. R. (1996). Epidemiological study of blood pressure in domestic dogs. *J. Small Anim. Pract.* 37 116–125. 8683954 10.1111/j.1748-5827.1996.tb02358.x

[B8] Boiten F. A. (1998). The effects of emotional behaviour on components of the respiratory cycle. *Biol. Psychol.* 49 29–51. 10.1016/s0301-0511(98)00025-8 9792483

[B9] Boiten F. A. Frijda N. H. Wientjes C. J. E. (1994). Emotions and respiratory patterns: review and critical analysis. *Int. J. Psychophysiol.* 17 103–128. 10.1016/0167-8760(94)90027-2 7995774

[B10] Boon J. Wingfield W. E. Miller C. W. (1983). Echocardiographic indices in the normal dog. *Vet. Radiol.* 24 214–221. 10.1111/j.1740-8261.1983.tb00718.x

[B11] Bunford N. Andics A. Kis A. Miklósi Á. Gácsi M. (2017). Canis familiaris as a model for non-invasive comparative neuroscience. *Trends Neurosci.* 40 438–452. 10.1016/j.tins.2017.05.003 28571614

[B12] Bunford N. Reicher V. Kis A. Pogány Á. Gombos F. Bódizs R. (2018). Differences in pre-sleep activity and sleep location are associated with variability in daytime/nighttime sleep electrophysiology in the domestic dog. *Sci. Rep.* 8:7109. 10.1038/s41598-018-25546-x 29740040 PMC5940857

[B13] Busek P. Vañková J. Opavský J. Salinger J. Nevšímalová S. (2005). Spectral analysis of heart rate variability in sleep. *Physiol. Res.* 54 369–376. 15588154

[B14] Coccagna G. Scaglione C. (2003). “Cardiocirculatory disorders and sleep,” in *Sleep*, ed. Billiard M. (Boston, MA: Springer), 589–597. 10.1007/978-1-4615-0217-3_47

[B15] Coote J. H. (1982). Respiratory and circulatory control during sleep. *J. Exp. Biol.* 100 223–244. 6757369 10.1242/jeb.100.1.223

[B16] Craig L. Meyers-Manor J. E. Anders K. Sütterlin S. Miller H. (2017). The relationship between heart rate variability and canine aggression. *Appl. Anim. Behav. Sci.* 188 59–67. 10.1016/j.applanim.2016.12.015

[B17] Dauvilliers Y. Rompré S. Gagnon J. F. Vendette M. Petit D. Montplaisir J. (2007). REM sleep characteristics in narcolepsy and REM sleep behavior disorder. *Sleep* 30 844–849. 10.1093/sleep/30.7.844 17682654 PMC1978363

[B18] Dickerson L. W. Huang A. H. Thumher M. M. Nearing B. D. Verrier R. L. (1993). Relationship between coronary hemodynamic changes and the phasic events of rapid eye movement sleep. *Sleep* 16 550–557. 10.1093/sleep/16.6.550 8235240

[B19] Ferasin L. Ferasin H. Little C. J. L. (2010). Lack of correlation between canine heart rate and body size in veterinary clinical practice. *J. Small Anim. Pract.* 51 412–418. 10.1111/j.1748-5827.2010.00954.x 20553373

[B20] Friedmann E. (2006). “The animal-human bond: health and wellness,” in *Handbook on Animal-Assisted Therapy: Theoretical Foundations and Guidelines for Practice* ed. Fine A. H. (Boston, MA: Elsevier) 41–58. 10.1016/B978-012369484-3/50005-0

[B21] Gácsi M. Maros K. Sernkvist S. Faragó T. Miklósi Á. (2013). Human analogue safe haven effect of the owner: behavioural and heart rate response to stressful social stimuli in dogs. *PLoS One* 8:e58475. 10.1371/journal.pone.0058475 23469283 PMC3587610

[B22] Gomez P. Danuser B. (2004). Affective and physiological responses to environmental noises and music. *Int. J. Psychophysiol.* 53 91–103. 10.1016/j.ijpsycho.2004.02.002 15210287

[B23] Hamlin R. L. Olsen I. Smith C. R. Boggs S. (1967). Clinical relevance of heart rate in the dog. *J. Am. Vet. Med. Assoc.* 151 60–63. 6068215

[B24] Hanton G. Rabemampianina Y. (2006). The electrocardiogram of the Beagle dog: reference values and effect of sex, genetic strain, body position and heart rate. *Lab. Anim.* 40 123–136. 10.1258/002367706776319088 16600072

[B25] Hezzell M. J. Humm K. Dennis S. G. Agee L. Boswood A. (2013). Relationships between heart rate and age, bodyweight and breed in 10,849 dogs. *J. Small Anim. Pract.* 54 318–324. 10.1111/jsap.12079 23662951

[B26] Hobson J. A. Goldfrank F. Snyder F. (1965). Respiration and mental activity in sleep. *J. Psychiatr. Res.* 3 79–90. 10.1016/0022-3956(65)90017-8 5825623

[B27] Homma I. Masaoka Y. (2008). Breathing rhythms and emotions. *Exp. Physiol.* 93 1011–1021. 10.1113/expphysiol.2008.042424 18487316

[B28] Hosey G. Melfi V. (2014). Human-animal interactions, relationships and bonds: a review and analysis of the literature. *Int. J. Comp. Psychol.* 27 117–142.

[B29] Ibáñez V. Silva J. Cauli O. (2018). A survey on sleep assessment methods. *PeerJ* 6:e4849. 10.7717/peerj.4849 29844990 PMC5971842

[B30] Katayama M. Kubo T. Mogi K. Ikeda K. Nagasawa M. Kikusui T. (2016). Heart rate variability predicts the emotional state in dogs. *Behav. Processes* 128 108–112. 10.1016/j.beproc.2016.04.015 27129806

[B31] Kis A. Gergely A. Galambos Á Abdai J. Gombos F. Bódizs R. (2017a). Sleep macrostructure is modulated by positive and negative social experience in adult pet dogs. *Proc. R. Soc. BBiol. Sci.* 284:20171883. 10.98/rspb.2017.1883 29070727 PMC5666109

[B32] Kis A. Szakadát S. Gácsi M. Kovács E. Simor P. Török C. S. (2017b). The interrelated effect of sleep and learning in dogs (Canis familiaris); an EEG and behavioural study. *Sci. Rep.* 7:41873. 10.1038/srep41873 28165489 PMC5292958

[B33] Kis A. Szakadát S. Kovács E. Gácsi M. Simor P. Gombos F. (2014). Development of a non-invasive polysomnography technique for dogs (Canis familiaris). *Physiol. Behav.* 130 149–156. 10.1016/j.physbeh.2014.04.004 24726397

[B34] Kittleson M. D. Kienle R. D. (1998). *Small Animal Cardiovascular Medicine.* St. Louis, MO: Mosby.

[B35] Kuhne F. Hößler J. C. Struwe R. (2014a). Behavioral and cardiac responses by dogs to physical human–dog contact. *J. Vet. Behav.* 9 93–97. 10.1016/j.jveb.2014.02.006

[B36] Kuhne F. Hößler J. C. Struwe R. (2014b). Emotions in dogs being petted by a familiar or unfamiliar person: validating behavioural indicators of emotional states using heart rate variability. *Appl. Anim. Behav. Sci.* 161 113–120. 10.1016/j.applanim.2014.09.020

[B37] Lamb A. P. Meurs K. M. Hamlin R. L. (2010). Correlation of heart rate to body weight in apparently normal dogs. *J. Vet. Cardiol.* 12 107–110. 10.1016/j.jvc.2010.04.001 20634163

[B38] Lanfranchi P. A. Fradette L. Gagnon J. F. Colombo R. Montplaisir J. (2007). Cardiac autonomic regulation during sleep in idiopathic REM sleep behavior disorder. *Sleep* 30 1019–1025. 10.1093/sleep/30.8.1019 17702272 PMC1978378

[B39] Lindstrom M. J. Bates D. M. (1990). Nonlinear mixed effects models for repeated measures data. *Biometrics* 46 673–687. 2242409

[B40] Lombard C. W. (1984). Normal values of the canine M-mode echocardiogram. *Am. J. Vet. Res.* 45 2015–2018. 6497098

[B41] Malik M. Bigger J. T. Camm A. J. Kleiger R. E. Malliani A. Moss A. J. (1996). Heart rate variability: standards of measurement, physiological interpretation, and clinical use. *Eur. Heart J.* 17 354–381. 10.1093/oxfordjournals.eurheartj.a014868 8737210

[B42] Maros K. Dóka A. Miklósi Á. (2008). Behavioural correlation of heart rate changes in family dogs. *Appl. Anim. Behav. Sci.* 109 329–341. 10.1016/j.applanim.2007.03.005

[B43] Masaoka Y. Homma I. (1997). Anxiety and respiratory patterns: their relationship during mental stress and physical load. *Int. J. Psychophysiol.* 27 153–159. 10.1016/s0167-8760(97)00052-4 9342646

[B44] Mather M. Thayer J. F. (2018). How heart rate variability affects emotion regulation brain networks. *Curr. Opin. Behav. Sci.* 19 98–104. 10.1016/j.cobeha.2017.12.017 29333483 PMC5761738

[B45] Miklósi Á Topál J. (2013). What does it take to become ‘best friends’? Evolutionary changes in canine social competence. *Trends Cogn. Sci.* 17 287–294. 10.1016/j.tics.2013.04.005 23643552

[B46] Miklósi Á. (2008). *Dog Behaviour, Evolution, and Cognition*, 1st editio Edn New York, NY: Oxford University Press.

[B47] Montano N. Porta A. Cogliati C. Costantino G. Tobaldini E. Casali K. R. (2009). Heart rate variability explored in the frequency domain: a tool to investigate the link between heart and behavior. *Neurosci. Biobehav. Rev.* 33 71–80. 10.1016/j.neubiorev.2008.07.006 18706440

[B48] Nyklíček I. Thayer J. F. Van Doornen L. J. (1997). Cardiorespiratory differentiation of musically-induced emotions. *J. Psychophysiol.* 11 304–321.

[B49] Orem J. O. H. N. (1980). Medullary respiratory neuron activity: relationship to tonic and phasic REM sleep. *J. Appl. Physiol.* 48 54–65. 10.1152/jappl.1980.48.1.54 7353979

[B50] Phillipson E. A. Murphy E. Kozar L. F. (1976). Regulation of respiration in sleeping dogs. *J. Appl. Physiol.* 40 688–693. 10.1152/jappl.1976.40.5.688 931895

[B51] Quintana D. S. Guastella A. J. Outhred T. Hickie I. B. Kemp A. H. (2012). Heart rate variability is associated with emotion recognition: direct evidence for a relationship between the autonomic nervous system and social cognition. *Int. J. Psychophysiol.* 86 168–172. 10.1016/j.ijpsycho.2012.08.012 22940643

[B52] Raetz S. L. Richard C. A. Garfinkel A. Harper R. M. (1991). Dynamic characteristics of cardiac RR intervals during sleep and waking states. *Sleep* 14 526–533. 10.1093/sleep/14.6.526 1798886

[B53] Rechtschaffen A. Kales A. (1968). *A Manual of Standardized Terminology, Techniques and Scoring Systemfor Sleep Stages of Human Subjects.* (Los Angeles: UCLA Brain Information Service, Brain Research Institute).

[B54] Rishniw M. Ljungvall I. Porciello F. Häggström J. Ohad D. G. (2012). Sleeping respiratory rates in apparently healthy adult dogs. *Res. Vet. Sci.* 93 965–969. 10.1016/j.rvsc.2011.12.014 22240295

[B55] Rowe K. Moreno R. Lau T. R. Wallooppillai U. Nearing B. D. Kocsis B. (1999). Heart rate surges during REM sleep are associated with theta rhythm and PGO activity in cats. *Am. J. Physiol.* 277 R843–R849. 10.1152/ajpregu.1999.277.3.R843 10484502

[B56] Saper C. B. Scammell T. E. Lu J. (2005). Hypothalamic regulation of sleep and circadian rhythms. *Nature* 437:1257. 10.1038/nature04284 16251950

[B57] Savolainen P. Zhang Y. P. Luo J. Lundeberg J. Leitner T. (2002). Genetic evidence for an east asian origin of domestic dogs. *Science* 298 1610–1613. 10.1126/science.1073906 12446907

[B58] Silvetti M. S. Drago F. Ragonese P. (2001). Heart rate variability in healthy children and adolescents is partially related to age and gender. *Int. J. Cardiol.* 81 169–174. 10.1016/s0167-5273(01)00537-x 11744133

[B59] Snyder F. Hobson J. A. Morrison D. F. Goldfrank F. (1964). Changes in respiration, heart rate, and systolic blood pressure in human sleep. *J. Appl. Physiol.* 19 417–422. 10.1152/jappl.1964.19.3.417 14174589

[B60] Somers V. K. Dyken M. E. Mark A. L. Abboud F. M. (1993). Sympathetic-nerve activity during sleep in normal subjects. *New Engl. J. Med.* 328 303–307. 10.1056/nejm199302043280502 8419815

[B61] Sztajzel J. (2004). Heart rate variability: a noninvasive electrocardiographic method to measure the autonomic nervous system. *Swiss Med. Weekly* 134 514–522. 15517504 10.4414/smw.2004.10321

[B62] Takeuchi T. Harada E. (2002). Age-related changes in sleep-wake rhythm in dog. *Behav. Brain Res.* 136 193–199. 10.1016/s0166-4328(02)00123-7 12385805

[B63] Tilley L. P. (1992). “Analysis of canine P-QRS-T deflections,” in *Essentials of Canine and Feline Electrocardiography*, ed. Tilley L. P. (Philadelphia, PA: Lea and Febigerp).

[B64] Tobaldini E. Nobili L. Strada S. Casali K. R. Braghiroli A. Montano N. (2013). Heart rate variability in normal and pathological sleep. *Front. Physiol.* 4:294. 10.3389/fphys.2013.00294 24137133 PMC3797399

[B65] Togo F. Yamamoto Y. (2001). Decreased fractal component of human heart rate variability during non-REM sleep. *Am. J. Physiol.* 280 H17–H21. 11123213 10.1152/ajpheart.2001.280.1.H17

[B66] Travain T. Colombo E. S. Grandi L. C. Heinzl E. Pelosi A. Previde E. P. (2016). How good is this food? A study on dogs’ emotional responses to a potentially pleasant event using infrared thermography. *Physiol. Behav.* 159 80–87. 10.1016/j.physbeh.2016.03.019 26996276

[B67] Trinder J. Kleiman J. Carrington M. Smith S. Breen S. Tan N. (2001). Autonomic activity during human sleep as a function of time and sleep stage. *J. Sleep Res.* 10 253–264. 10.1046/j.1365-2869.2001.00263.x 11903855

[B68] Varga B. Gergely A. Galambos Á. Kis A. (2018). Heart rate and heart rate variability during sleep in family dogs (*Canis familiaris*). Moderate effect of pre-sleep emotions. *Animals* 8:107. 10.3390/ani8070107 30004461 PMC6071078

[B69] Vaughn B. V. Quint S. R. Messenheimer J. A. Robertson K. R. (1995). Heart period variability in sleep. *Electroencephalogr. Clin. Neurophysiol.* 94 155–162. 7536150 10.1016/0013-4694(94)00270-u

[B70] Verrier R. L. Lau T. R. Wallooppillai U. Quattrochi J. Nearing B. D. Moreno R. (1998). Primary vagally mediated decelerations in heart rate during tonic rapid eye movement sleep in cats. *Am. J. Physiol.* 274 R1136–R1141. 10.1152/ajpregu.1998.274.4.R1136 9575980

[B71] von Borell E. Langbein J. Després G. Hansen S. Leterrier C. Marchant-Forde J. (2007). Heart rate variability as a measure of autonomic regulation of cardiac activity for assessing stress and welfare in farm animals—a review. *Physiol. Behav.* 92 293–316. 10.1016/j.physbeh.2007.01.007 17320122

[B72] Zupan M. Buskas J. Altimiras J. Keeling L. J. (2016). Assessing positive emotional states in dogs using heart rate and heart rate variability. *Physiol. Behav*. 155, 102–111. 10.1016/j.physbeh.2015.11.027 26631546

